# IS*15DIV*-flanked composite transposon harboring *bla*_NDM-5_ in multidrug-resistant *Salmonella* Typhimurium

**DOI:** 10.1016/j.isci.2024.111720

**Published:** 2024-12-31

**Authors:** Kaiting Zhao, Jing Jin, Yuan Liao, Aixia Liu, Wugao Liu, Weiping Wu

**Affiliations:** 1 Department of Clinical Laboratory, Lishui People’s Hospital, The Sixth Affiliated Hospital of Wenzhou Medical University, Lishui, Zhejiang, China; 2 Department of Hematology, Lishui People’s Hospital, The Sixth Affiliated Hospital of Wenzhou Medical University, Lishui, Zhejiang, China

**Keywords:** Multidrug-resistant organisms, Biochemistry, Microbiology

## Abstract

Multidrug-resistant *Salmonella* Typhimurium has emerged as a global public health concern. Asymptomatic gastrointestinal carriage is a key factor in the spread of antibiotic-resistant bacteria. However, it is challenging to obtain direct evidence of *in vivo* transfer of mobile genetic elements (MGEs). Here, we found that MGEs harboring *bla*_NDM-5_ were transferred from asymptomatic *Escherichia coli* (LS20223694) to *Salmonella* Typhimurium (LS20223695) in a child. BLAST analyses demonstrated that the IS*15DIV*-flanked composite transposon of pLS20223695_NDM5 showed high similarity with pLS20223694_NDM5 and two previously reported plasmids, suggesting the possibility of genetic recombination. Besides, conjugation experiments showed that the transconjugant carried the incompatibility group I1 (IncI1)-I(alpha) plasmid replicon with the *bla*_NDM-5_ and *sul1* genes, indicating that pLS20223695_NDM5 is a conjugative plasmid with transferability. Our study provides insights into the genetic basis of an IS*15DIV*-flanked composite transposon in *Salmonella* Typhimurium.

## Highlights

• Carbapenem-resistant *S.* Typhimurium and *E. coli* isolated from the same patient
• MGEs harboring *bla*_NDM-5_ were transferred from *E. coli* to *S.* Typhimurium
• IS*15DIV*-flanked composite transposon in MGEs of *S.* Typhimurium and *E. coli*
• Transferability of plasmids in *S.* Typhimurium and *E. coli*

## Graphical abstract

**Figure:**
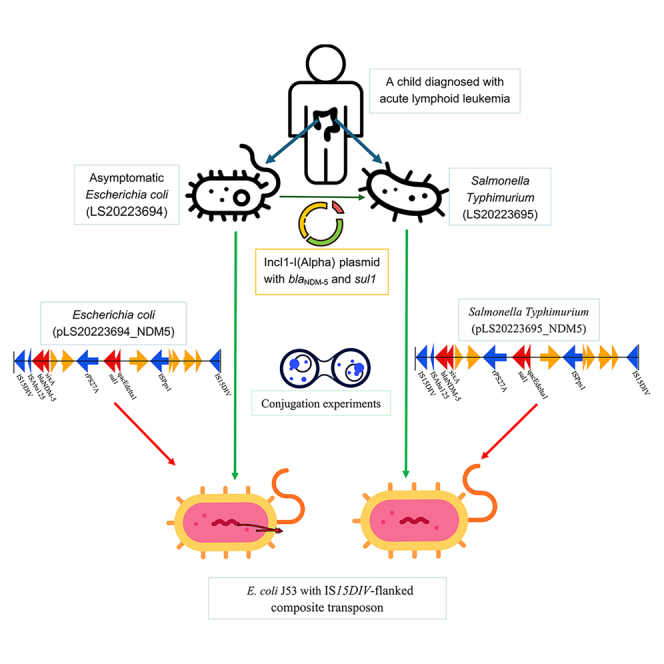

## Introduction

*Salmonella* is a foodborne zoonotic pathogen that threatens human health, livestock production, and food safety.^1^ *Salmonella* Typhimurium (*S.* Typhimurium) is one of the most prevalent serotypes causing gastroenteritis or invasive disease in humans, especially immunocompromised individuals (such as people with HIV infection, hematopathy, and malnutrition), young children, and the elderly individuals.^2^ Multidrug-resistant *Salmonella* strains, particularly those resistant to carbapenems, have exacerbated the global challenge of establishing effective treatment and management policies.^3^ Twenty-nine variants of the New Delhi metallo-β-lactamase (NDM) gene have been identified in bacteria worldwide since 2009.^4^ Among these variants, NDM-5 stands out as one of the most prevalent among *Enterobacteriaceae*, posing a significant challenge due to the limited availability of effective antibiotics.^5^ While reports of carbapenem-resistant *Enterobacteriaceae* (CRE) carrying *bla*_NDM-5_ have garnered widespread attention from various regions,^6^^,^^7^ their genetic backgrounds remain highly conserved, often residing on MGEs, facilitating their rapid dissemination.^8^ According to whole-genome sequencing (WGS) data of global carbapenem-resistant *Salmonella enterica* (CRSE) isolates, *S.* Typhimurium (21.8%) was the most prevalent CRSE serotypes worldwide, and *bla*_NDM_ (65.7%) was predominant in Asian CRSE.^9^ However, NDM-5-producing *S.* Typhimurium is still rare.

The involvement of MGEs, such as insertion sequences and transposons, plays a critical role in enhancing the adaptability and survival of CRE. These elements are essential for the homologous recombination processes that facilitate the acquisition and dissemination of antimicrobial resistance genes, leading to the reshuffling of plasmid structures and influencing the fitness of bacterial pathogens.^10^^,^^11^ Insertion sequences are small MGEs that are characterized as containing only the functions required for self-mobilization. Notably, the insertion sequence (IS) IS*26* plays a key role in catalyzing the formation of composite transposons containing multiple resistance genes.^12^ Worse still, IS*26*-related transposons have been shown to produce unexpected phenotypes during antibiotic treatment.^13^^,^^14^^,^^15^ For instance, IS*26*-mediated amplification of *aphA1* gene leads to tobramycin resistance in *Acinetobacter baumannii*.^14^ Therefore, exploring the dynamics of MGEs within plasmids is crucial for understanding and combating infections associated with clinical multidrug-resistant strains carrying *bla*_NDM-5_.

Asymptomatic gastrointestinal carriage is a key factor in the spread and epidemiology of antibiotic-resistant bacteria. The emergence of antibiotic resistance genes in human intestinal bacteria occurred via mobilization from such an antibiotic gene pool either through chromosomal mutations or mainly by intraspecies or interspecies horizontal transfer of plasmids or MGEs.^16^^,^^17^^,^^18^ However, it is challenging to obtain direct evidence of *in vivo* transfer, as it is difficult to pinpoint the original donor strain responsible for transferring a new resistance gene to a recipient strain. Here, we characterized two CRE species from the fecal sample of same patient and found the plasmid harboring *bla*_NDM-5_ transferred between *Escherichia coli* (*E. coli*) and *S.* Typhimurium.

## Results

### Phenotypic characterization of *S.* Typhimurium and *E. coli*

The *S.* Typhimurium LS20223695 (BioSample ID: SAMN41075555) genome (136,615 reads, contig N50: 11,519 bp) contains a 4,952,660 bp circular chromosome with an average GC content of 52.12% and a 289,173 bp plasmid. The *E. coli* LS20223694 (BioSample ID: SAMN42812417) genome (196,593 reads, contig N50: 11,550 bp) contains a 5,108,357 bp circular chromosome with an average GC content of 50.74% and two plasmids (152 and 41 bp). Meanwhile, these strains were found to be resistant to all of the antibiotics tested except for aztreonam, including ceftriaxone (CRO), ceftazidime (CAZ), cefepime, amikacin (AK), cefoxitin (FOX), amoxicillin/clavulanic acid (AMC), piperacillin/tazobactam (TZP), levofloxacin (LEV), trimethoprim/sulfamethoxazole (SXT), cefuroxime (CXM), imipenem (IPM), ertapenem (ETP), and cefoperazone/sulbactam (SCF) (Table 1). In accordance with the resistance phenotype, the genomic analysis revealed that *S.* Typhimurium LS20223695 harbored *bla*_NDM-5_, *bla*_OXA-1_, *aac(6′)-Ib-cr*, *aph(4)-Ia*, *aadA2*, *sul1*, *floR*, *catB3*, *oqxB*, *tet(B)*, *dfrA12*, and *bleO*, which may confer resistance to carbapenems, aminoglycosides, sulfonamide, chloramphenicol, fluoroquinolone, tetracycline, trimethoprim, and other β-lactams. Moreover, the following resistance genes were identified in *E. coli* LS20223694: *bla*_NDM-5_, *bla*_CTX-M-15_, *aac(3)-IIa*, *aph(6)-Id*, *aadA5*, *sul1*, *mph(A)*, *erm(B)*, *floR*, *tet(A)*, and *dfrA17*.

**Table 1:** Characteristics of different isolates and their corresponding transconjugants

Isolate	*bla*_*NDM-5*_ location	Antibiotic minimum inhibitory concentration (μg/mL)													
		CRO	CAZ	FEP	AK	FOX	AMC	TZP	LEV	SXT	CXM	ATM	IPM	ETP	SCF
LS20223694	152-kb plasmid	≥64	≥64	≥32	≤2	≥64	≥32	≥128	≥8	≥320	≥64	2	≥16	≥8	≥64
LS20223695	289-kb plasmid	≥64	≥64	16	8	≥64	≥32	≥128	2	≥320	≥64	2	≥16	≥8	≥64
LS20223694-TC	94-kb plasmid	≥64	≥64	16	≤2	≥64	≥32	≥128	≤0.12	≤20	≥64	2	≥16	≥8	≥64
LS20223695-TC	109-kb plasmid	≥64	≥64	2	≤2	≥64	≥32	≥128	≤0.12	≤21	≥64	2	8	≥8	≥64
*E. coli* J53	–	≤0.25	≤0.12	2	≤2	≤4	4	≤4	≤0.12	≤20	4	2	≤0.25	≤0.12	≤8

TC, corresponding transconjugant; CRO, ceftriaxone; CAZ, ceftazidime; FEP, cefepime; AK, amikacin; FOX, cefoxitin; AMC, amoxicillin/clavulanic acid; TZP, piperacillin/tazobactam; LEV, levofloxacin; SXT, trimethoprim/sulfamethoxazole; CXM, cefuroxime; ATM, aztreonam; IPM, imipenem; ETP, ertapenem; SCF, cefoperazone/sulbactam.

### cgMLST and core SNP phylogenetic tree

**Figure 1:**
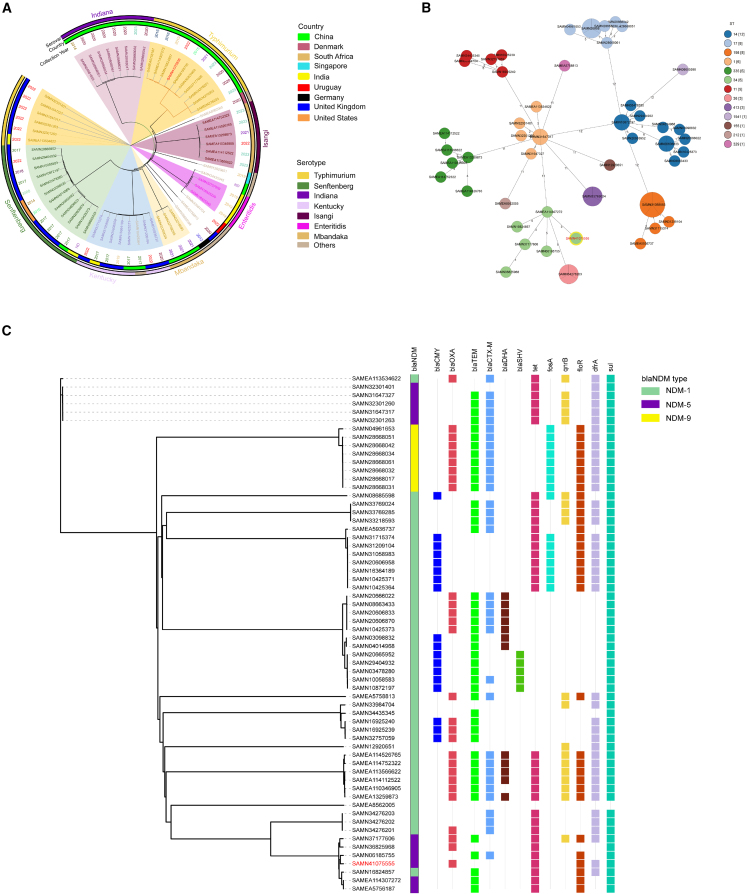
Phylogeny of global NDM-CRSE isolates (61 NDM-CRSE strains retrieved from GenBank database and *S.* Typhimurium LS20223695 [BioSample ID: SAMN41075555]) recovered from this study (A) The SNP tree of NDM-CRSE isolates based on the isolation countries, serotypes, and isolation years. (B) The MST analysis of NDM-CRSE isolates. The numbers on the branches represent the number of loci that differ between isolates. Node color coding represents the STs of the isolates. (C) Binary heatmap analysis of antimicrobial resistance genes harbored by the NDM-CRSE isolates. SAMN41075555 is highlighted in red. NDM-CRSE, carbapenem-resistant *Salmonella enterica* harboring *bla*_NDM_; ST, sequence type.

According to the WGS data of global CRSE harboring *bla*_NDM_ (NDM-CRSE) isolates, 40.3% of the isolates were predominant in China. Typhimurium (25.8%) and Senftenberg (19.4%) were the most prevalent isolates (Figure 1A). *In silico* multi-locus sequence typing results showed that sequence type ST14 (19.4%) was predominantly found in NDM-CRSE isolates and *S.* Typhimurium LS20223695 belonged to ST34 (Figure 1B). The most closely related strain of *S.* Typhimurium LS20223695 was SAMEA114307272 from China in 2016 with 5 allele differences. Additionally, 71.0% of the NDM-CRSE isolates carried the NDM-1 gene and 80.6% carried extended-spectrum β-lactamase (ESBL) genes, primarily *bla*_TEM_ (76.7%), as well as high resistance rates to sulfonamides (98.4%), trimethoprim (67.7%), tetracycline (53.2%), and chloramphenicol (53.2%) (Figure 1C). Interestingly, similar to *S.* Typhimurium LS20223695, most of NDM-CRSE (53.2%) strains carried the IS*26*-*bla*_NDM_-IS*26* composite transposon, indicating that the IS*26*-flanked composite transposon was conserved in most NDM-CRSE genomes in the GenBank (Figure S1).

### Genomic features of plasmids harboring the *bla*_NDM-5_ gene in *S.* Typhimurium and *E. coli*

**Figure 2:**
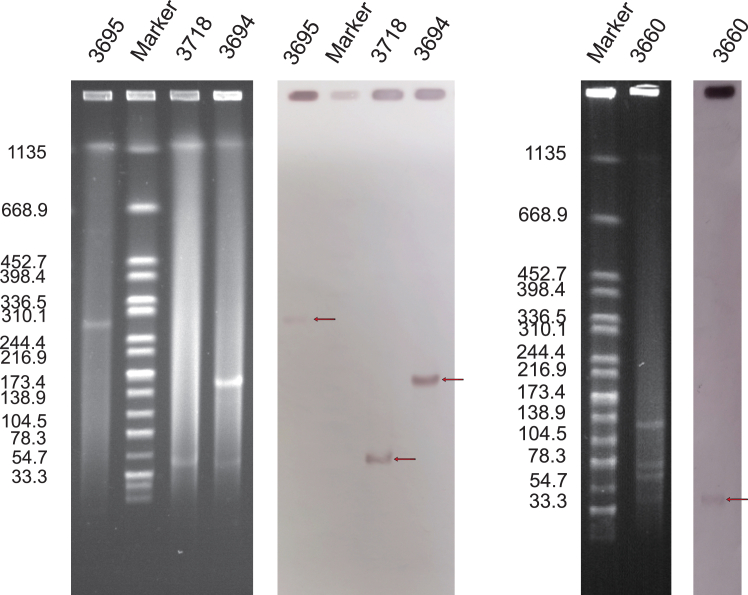
S1-PFGE and Southern blot hybridization results for the *S.* Typhimurium strain LS20223695 and *E.* coli strains LS20223694, LS20023660, and LS20023718, which were derived from the Lishui People’s Hospital The arrow depicts the position of the plasmid band in which a positive hybrid semaphore of the *bla*_NDM-5_ gene was detected.

The pLS20223694_NDM5 plasmid is 152,131 bp in length and has a GC content of 50.8%. The pLS20223695_NDM5 plasmid is 289,173 bp in length and has a GC content of 47.7%. Consistent with the WGS results, S1-PFGE illustrated that *S.* Typhimurium LS20223695 contained a single plasmid of ∼289 kb, and *E.* coli LS20223694 contained two plasmids, the sizes of which were found to be ∼41 and ∼152 kb. Meanwhile, we retrospectively screened 8 carbapenem-resistant *E. coli* strains from the hematology department between June 2022 and June 2023 for the presence of a plasmid carrying the NDM-5 gene. Two *bla*_NDM5_-positive *E. coli* strains (LS2023660 and LS2023718) were screened in the department. Southern hybridization suggested that the *bla*_NDM-5_ gene was located in pLS20223695_NDM5 (289 kb), pLS20223694_NDM5 (152 kb), pLS20023660 (44 kb), and pLS20023718 (46 kb) (Figure 2).

**Figure 3:**
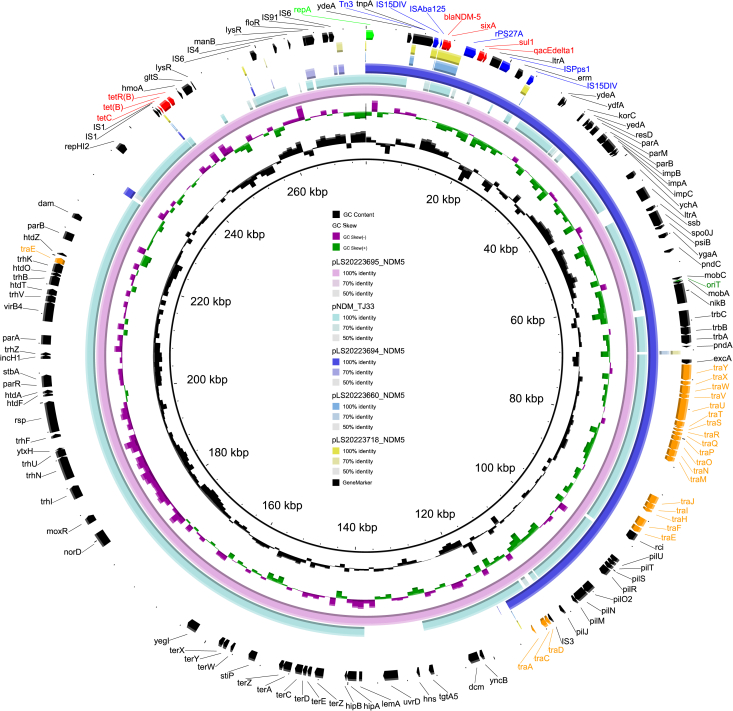
Genetic organization of plasmids harboring *bla*_NDM-5_ Circular alignment of plasmids pLS20223695_NDM5, pLS20223694_NDM5, pLS20023660, and pLS20023718 with similar plasmids in the NCBI GenBank database. The comparisons were obtained relative to the innermost circle (pLS20223695_NDM5). The outermost ring indicates genes as arrows in the corresponding transcription orientation as follows: red, resistance genes; blue, mobile genetic elements; orange, transfers genes; black, hypothetical proteins. The origin of transfer site (*ori*T) is also indicated.

The backbone of pLS20223695_NDM5 and pLS20223694_NDM5 harbored replication gene (*repA*), resistance genes, mobile genetic elements, transfer genes, and origin of transfer site (*oriT*) (Figure 3). BLAST analysis showed that the backbone of pLS20223695_NDM5 exhibited high homology (63% coverage and 100% identity) to the plasmid pNDM-TJ33 (BioProject: PRJNA224116). Interestingly, all of these plasmids contained the sequence of the transposon (IS*15DIV*-IS*Aba125*-*bla*_NDM-5_-*sixA*-IS*15DIV*), suggesting that this transposon might be acquired through the transmission of plasmids harboring *bla*_NDM-5_. In addition, the plasmids pLS20023660 and pLS20023718 exhibited very low homology to pLS20223694_NDM5, indicating that the *bla*_NDM-5_-positive plasmid of LS20223694 might not have originated from pathogens in the hematology department.

**Figure 4:**
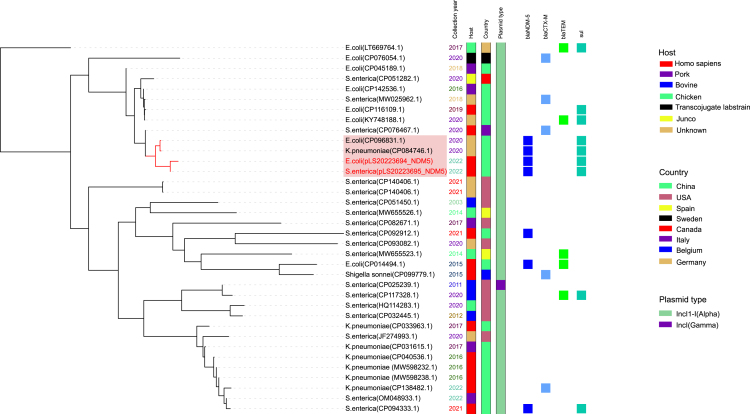
Phylogenetic analysis of pLS20223695_NDM5, pLS20223694_NDM5, and 34 publicly available similar plasmids in the NCBI GenBank database Different colors are used to indicate the collection years, hosts, countries, plasmid types, and antimicrobial resistance profiles. The plasmid sequences (pLS20223695_NDM5 and pLS20223694_NDM5) from this study are highlighted in red color.

**Figure 5:**
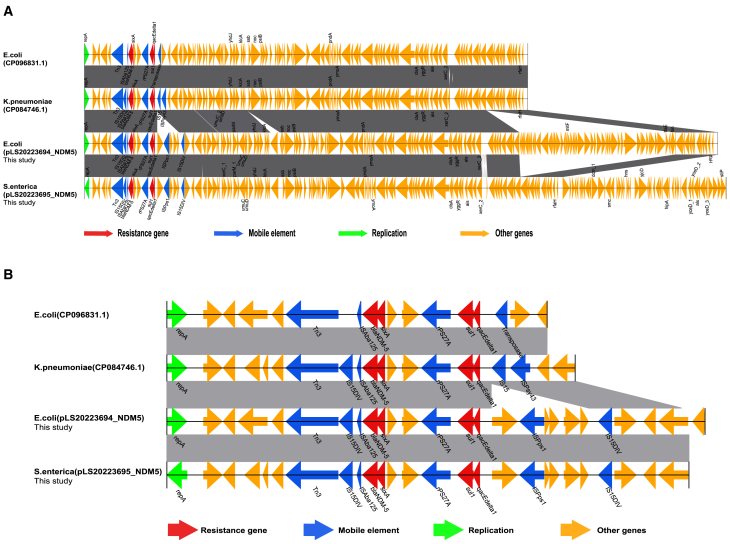
Linear comparison of plasmid pLS20223695_NDM5 with other similar plasmids with *bla*_NDM-5_ (A) Alignment of plasmid pLS20223695_NDM5 in *S.* Typhimurium and plasmid pLS20223694_NDM5 in *E. coli* recovered from this study with plasmids deposited in NCBI database, including *E. coli* (CP096831.1) and *K*. *pneumoniae* (CP084746.1). (B) Linear comparison of the MDR regions of pLS20223695_NDM5 and pLS20223694_NDM5 with those of *E. coli* (CP096831.1) and *K*. *pneumoniae* (CP084746.1). The gray shading indicates regions of shared homology among different elements. The open reading frames are marked by colored arrows, red indicates resistance genes, blue indicates mobile elements, green indicates genes encoding plasmid replicons, and orange indicates other genes.

Phylogenetic analysis of pLS20223695_NDM5, pLS20223694_NDM5, and 34 publicly available similar plasmids in the NCBI GenBank database, including samples from six hosts, eight countries, and two plasmid types from 2003 to 2022, most of which belong to the incompatibility group I1 (IncI1)-I(alpha) (97.2%) replicon types. The main isolation country was China (19/36, 52.8%), of which 68.4% were originated from clinical samples, indicating the horizontal transmission of similar plasmids among *Enterobacteriaceae* of inpatients (see Figure 4). Moreover, pLS20223695_NDM5, pLS20223694_NDM5, and *E*. coli (CP096831.1) along with *K*. *pneumoniae* (CP084746.1) showed a high degree of genetic similarity, and all of them emerged in China and persisted in one cluster. As shown in Figure 5A, the sequence of pLS20223695_NDM5 showed 100% coverage and 100% identity with the pLS20223694_NDM5, featuring identical resistance genes and IS. In addition, the pLS20223695_NDM5 exhibited 93% coverage and 100% identity with *E.* coli (CP096831.1) and showed 64% coverage and 100% identity with *K. pneumoniae* (CP084746.1). BLAST comparison showed high similarity between the multidrug resistance (MDR) region (IS*15DIV*-IS*Aba125*-*bla*_NDM-5_-*sixA-rPS27A-sul1-qacEdelta1*-IS*Pps1*-IS*15DIV*) of these plasmids (Figure 5B).

### Conjugation experiments

Compared with those of the recipient *E. coli* J53, the minimum inhibitory concentrations of CRO, CAZ, FOX, AMC, TZP, CXM, IPM, ETP, and SCF of the transconjugants LS20223694-TC and LS20223695-TC were higher. Moreover, all the transconjugants remained susceptible to AK, LEV, and SXT (Table 1). The conjugation rates were observed in the LS20223694-TC and LS20223695-TC at 6.92 × 10^−5^ and 3.04 × 10^−5^, respectively. The sequencing results showed that the transconjugants carried IncI1-I(alpha) plasmids with *bla*_NDM-5_ and sul1 genes, which indicates that the plasmids pLS20223695_NDM5 and pLS20223694_NDM5 carrying the *bla*_NDM-5_ genes are conjugative plasmids with transferability.

## Discussion

*S*. Typhimurium has emerged as a global public health concern in the last two decades, largely because of its ability to exhibit multidrug resistance.^19^ This strain has undergone a notable increase in its isolation from clinical specimens and various animal reservoirs, particularly from food-producing animals and food products from Asia to North America,^20^ and has risen to one of the primary *Salmonella* serotypes responsible for both human and animal infections in Europe and the United States,^21^ highlighting the rapid dissemination of this MDR serotypes worldwide. This escalating prevalence underscores the urgent need for heightened vigilance and global cooperation to mitigate its impact on public health.

Carbapenem resistance in *Enterobacteriaceae* often involves various mechanisms such as the production of carbapenemases, the loss of outer membrane porins, and increased efflux pump activity.^22^^,^^23^ Various CRSE strains have been documented globally, encompassing KPC-2,^24^ NDM-1,^25^ and VIM-1.^26^ Carbapenem-resistant *S.* Typhimurium is a significant clinical concern because of the limited treatment options. In 2015, the first *bla*_NDM-5_-positive IncFII plasmid isolated from *S.* Typhimurium ST34 was found in a child with acute diarrhea from Guangdong, China.^27^ However, *bla*_NDM-5_-positive plasmid is still rarely reported in *S*. Typhimurium. In this study, we performed genetic characterization of *S.* Typhimurium LS20223695, which carries the *bla*_NDM-5_-positive IncI1-I(alpha) plasmid, and figured out whether its plasmid can be successfully transferred into *E. coli* J53.

Two years ago, *S*. Typhimurium ST34 drew attention from global public health authorities because of causing infections in 143 children under the age of ten in at least 11 countries.^28^ In this study, *S.* Typhimurium LS20223695 belonged to ST34. Consistent with a previous study on the global CRSE,^9^ we found that almost all the ST34-type NDM-CRSE isolates were *S.* Typhimurium and originated from China. However, it is worth noting that some pairs originating from different countries are well below the threshold of clonal transmission; for instance, strain SAMN31058983 from India differs by only 1 allele compared with strain SAMN31209104 from the UK. Similarly, compared with SAMN08663433 from India, strains SAMN20606833 from the UK and SAMN10425373 from China showed differences of merely 1 allele each. This phenomenon suggests that NDM-CRSE may facilitate cross-border transmission, potentially leading to global dissemination. Additionally, 71.0% of the NDM-CRSE isolates carried the NDM-1 gene, and 80.6% carried ESBL genes, primarily *bla*_TEM_ (76.7%), as well as high resistance rates to sulfonamides (98.4%). However, only 34.5% of the CRSE strains carried ESBL genes, and resistance rates to sulfonamides (75.9%) were reported in a previous study, indicating that the NDM-CRSE has developed into pandrug-resistant bacteria more easily.

Asymptomatic gastrointestinal carriage of CRE can precede infections, potentially turning carriers into focal points for transmission among patients and healthcare personnel, facilitating the spread of clonal and plasmid-mediated resistance traits.^29^ Screening stool samples for CRE enables clinicians to identify the presence of multidrug-resistant *Enterobacteriaceae* colonizing the gut in patients vulnerable to potential endogenous infections. Such screening efforts are crucial for establishing enhanced systems to control CRE transmission and infection within hospital settings. In this study, we identified a CRSE harboring *bla*_NDM-5_ from a child who had multiple visits to the hospital over several years since being diagnosed with acute lymphoid leukemia in April 2020. He was treated with piperacillin-tazobactam in March 2022. Two months later, this patient was treated with IPM-cilastatin because of fever. Six hours later, the *E. coli* strain was isolated from the fecal sample when it was subjected to CRE screening upon admission. However, the *S.* Typhimurium strain was not detected at admission. In June 2022, he experienced fever and acute diarrhea before admission. The carbapenem-resistant *E. coli* and *S.* Typhimurium isolates were discovered from a stool sample simultaneously. We speculate that a plasmid harboring *bla*_NDM-5_ was transferred from *E. coli* LS20223694 to *S.* Typhimurium LS20223695.

The mobility of *bla*_NDM_ involves diverse processes such as genetic recombination, conjugation and transformation of plasmids, transduction, and transfer through outer membrane vesicles.^30^ Many mobile elements play crucial roles in dissemination, including IS*26*, IS*Aba125*, IS*5*, IS*CR1*, Tn*3*, Tn*125*, and Tn*3000*.^31^^,^^32^ The unique role of the IS in facilitating recombination events and extensive transfer of resistance genes is well known.^33^ While IS elements from other families, such as IS*1*, IS*10*, IS*Ecp1*, and IS*Apl1*, play key roles in mobilizing antibiotic resistance genes in gram-negative bacteria,^34^ IS*26* stands out as the predominant contributor. When two IS*26* elements are arranged in the same or opposite directions, they often combine to form a composite transposon, facilitating the transfer of resistance genes between them.^30^ There are several minor variants of IS*26* (such as IS*15* or IS*15DIV*) that exhibit slight variations at one or a few positions, resulting in single amino acid differences in the transposase.^13^ Moreover, NDM-positive isolates consistently carry either a complete or fragmented IS*Aba125* across multiple species, upstream of *bla*_NDM_, which provides a promoter region.^35^^,^^36^^,^^37^ In this study, the sequence of the transposon (IS*15DIV*-IS*Aba125*-*bla*_NDM-5_-*sixA-rPS27A-sul1-qacEdelta1*-IS*Pps1*-IS*15DIV*) from transconjugants LS20223695-TC and LS20223694-TC showed 100% coverage and 100% identity with the pLS20223695_NDM5, indicating the critical role of IS*15DIV* and IS*Aba125* in the horizontal transmission of *bla*_NDM-5_ and other resistance determinants. Importantly, our study identified *bla*_NDM-5_ and *sul1* in LS20223695-TC and LS20223694-TC, indicating that *bla*_NDM-5_ could co-transfer with *sul1* across different bacterial species, thereby promoting the spread of drug resistance.

### Conclusion

In this study, an IncI1-I(alpha) plasmid harboring *bla*_NDM-5_ was transferred from asymptomatic *E. coli* to *S.* Typhimurium, which isolated from a stool sample of a child diagnosed with acute lymphoid leukemia in Lishui, Zhejiang Province. The conjugation experiments showed that this IncI1-I(alpha) plasmid is a conjugative plasmid with transferability. The genetic arrangement of *bla*_NDM-5_, represented as “IS*15DIV*-IS*Aba125*-*bla*_NDM-5_-*sixA-rPS27A-sul1-qacEdelta1*-IS*Pps1*-IS*15DIV*,” deviates from previously documented structures, utilizing IS*15DIV* as composite transposons at both ends for gene transfer. Moreover, the co-transfer of *bla*_NDM-5_ and *sul1* demands increased attention, as it may accelerate the emergence of *S.* Typhimurium strains resistant to carbapenems and sulfonamides antibiotics.

### Limitations of the study

We acknowledge two limitations in our study. First, in our study, the time span and sample size were small, resulting in little information about changes in the timeline. Second, although we included all NDM-CRSE isolates from publicly available databases, we may still not reach a high-enough confidence level to infer the geographic spread and local evolution of NDM-CRSE around the world, which could affect the conclusions.

## Resource availability

### Lead contact

Further information and requests can be directed to the lead contact, Weiping Wu (wwp10145@163.com).

### Materials availability

This study did not generate new unique reagents.

### Data and code availability

• The genomes of *S.* Typhimurium LS20223695 (BioSample ID: SAMN41075555) and *E. coli* LS20223694 (BioSample ID: SAMN42812417) have been deposited at NCBI GenBank. Accession numbers are listed in the key resources table.
• This paper does not report original code.
• This paper does not report any other items.

## Acknowledgments

This project has received funding from the Science and Technology Planning Project of Lishui (2023GYX50).

## Author contributions

K.Z. and W.W. designed the experiments and wrote the manuscript. W.L., J.J., and Y.L. performed the experiments, library construction, and analyzed the data. A.L., W.L., and J.J. prepared the tables and figures. W.L. and W.W. finally revised the manuscript.

## Declaration of interests

The authors declare no competing interests.

## STAR★Methods

### Key resources table

**Table:** 

REAGENT or RESOURCE	SOURCE	IDENTIFIER
**Bacterial and virus strains**		

*Escherichia coli* (LS20223694)	This study	N/A
*Salmonella* Typhimurium (LS20223695)	This study	N/A
*Escherichia coli* (LS2023660)	This study	N/A
*Escherichia coli* (LS2023718)	This study	N/A
*Escherichia coli* J53	Institute of Antibiotics, Huashan Hospital, Shanghai, China	N/A

**Chemicals, peptides, and recombinant proteins**		

Luria-Bertani broth	Hangzhou Binhe Microorganism Reagent Co., Ltd, China	Cat# HTWS-B104
tryptic soy agar	Hangzhou Binhe Microorganism Reagent Co., Ltd, China	Cat# HTWS-B347
sodium azide	Sigma-Aldrich, shanghai, China	Cat# 26628-22-8
meropenem	Luoxin Pharmaceutical Group Stock Co., Ltd.,Shandong, China	Cat# 96036-03-2
S1-nuclease	Takara, Dalian, China	Cat# 2410A
gold agarose gel plugs	SeaKem® Gold Agarose, Lonza, USA	Cat# 50150

**Critical commercial assays**		

NovaSeq 6000	Illumina, California, USA	N/A
Nanopore	Oxford, UK	N/A
DIG High Prime DNA Labling and Detection Starter kit 1	Roche Diagnostics, Germany	Cat# 11745832910

**Deposited data**		

*Escherichia coli* (LS20223694)	This study	SAMN42812417
*Salmonella* Typhimurium (LS20223695)	This study	SAMN41075555
pNDM-TJ33	GenBank	BioProject: PRJNA224116
*E.* coli (CP096831.1)	GenBank	SAMN27593794
*K. pneumoniae* (CP084746.1)	GenBank	SAMN21849102

**Software and algorithms**		

Unicycler v.0.4.7	Wick et al.^19^	https://github.com/rrwick/Unicycler
Prokka v.1.14.6	Seemann et al.^38^^,^^39^	https://github.com/tseemann/prokka
ResFinder 4.6.0	Zankari et al.^40^^,^^41^	https://cge.food.dtu.dk/services/ResFinder/
BLAST Ring Image Generator (BRIG)	Alikhan et al.^20^	https://sourceforge.net/projects/brig/
NCBI RefSeq database	N/A	https://www.ncbi.nlm.nih.gov/refseq/
EnteroBase	Achtman et al.^21^	https://enterobase.warwick.ac.uk/
RAxML v8.2.9	Stamatakis et al.^42^	http://sco.h-its.org/exelixis/web/software/raxml/
OriTFinder	Li, X. et al.^43^	https://bioinfo-mml.sjtu.edu.cn/oriTfinder/
PlasmidFinder 2.1	Carattoli et al.^44^	https://cge.food.dtu.dk/services/PlasmidFinder/

### Experimental model and study participant details

In this study, *S.* Typhimurium LS20223695 and *E. coli* LS20223694 were isolated from the fecal sample of a 7-year-old boy who was hospitalized in the hematology department. Two *bla*_NDM5_-positive *E. coli* strains (LS2023660 and LS2023718) were screened from the fecal samples between June 2022 and June 2023 in the hematology department.

#### Ethics statements

The Ethics approval and a waiver of informed consent was granted by the Ethics Committee of the Lishui People’s Hospital (Issuing No. 2024-053), due to retrospective analysis of anonymized data.

### Method details

#### Bacterial isolation and phenotypic characterization

A 7-year-old child was hospitalized in the hematology department and diagnosed with acute lymphoid leukemia. *E. coli* strain (LS20223694) was isolated from the child’s fecal sample when undergoing CRE screening with the onset of fever and diarrhea. At the same time, the *S.* Typhimurium (LS20223695) was isolated from the fecal sample. The isolates were identified by matrix-assisted laser desorption time-of-flight mass spectrometry (MALDI-TOF MS) (Bruker Daltonik GmbH, Bremen, Germany) and serotyped with commercial antiserum (Tianrun, Ningbo, China). The minimum inhibitory concentrations (MICs) of ceftriaxone (CRO), ceftazidime (CAZ), cefepime (FEP), amikacin (AK), cefoxitin (FOX), amoxicillin/clavulanic acid (AMC), piperacillin/tazobactam (TZP), levofloxacin (LEV), trimethoprim/sulfamethoxazole (SXT), cefuroxime (CXM), aztreonam (ATM), imipenem (IMP), ertapenem (ETP), and cefoperazone/sulbactam (SCF) were determined by the VITEK 2 system (bioMérieux, Marcy-l’Étoile, France) and explained in agreement with the Clinical and Laboratory Standards Institute (CLSI M100, 33rd edition) guidelines. The quality control strain was *E. coli* ATCC 25922. We obtained permission to report the cases from the patients’ families.

#### Whole-genome sequencing and bioinformatics analysis

Whole-genome sequencing (WGS) was conducted by using the NovaSeq 6000 (Illumina, California, USA) and Nanopore (Nanopore, Oxford, UK) platforms at Novogene Bioinformatics Technology Co., Ltd (Tianjin, China). The derived short reads and long reads were assembled by using Unicycler v.0.4.7 software.^45^ The bacterial genomes annotated by using Prokka v.1.14.6.^38^ Antibiotic resistance genes were confirmed by using the ResFinder database (https://cge.food.dtu.dk/services/ResFinder/). The Inc groups of plasmids were analysed by PlasmidFinder (https://cge.food.dtu.dk/services/PlasmidFinder/). The origin of transfers in DNA sequences of bacterial mobile genetic elements were searched with OriTFinder (https://bioinfo-mml.sjtu.edu.cn/oriTfinder/). Sequence comparisons between plasmids harboring *bla*_NDM-5_ were performed via the BLAST Ring Image Generator (BRIG).^46^ Among a total of 82 *Salmonella* strains harboring NDM genes extracted from the NCBI RefSeq database (accessed 2nd April, 2024), the SRA sequences of 61 strains were identified. All 61 selected *Salmonella* strains and *S.* Typhimurium LS20223695 were analyzed via the EnteroBase website (https://enterobase.warwick.ac.uk/) basing on the Construction of Hierarchical Clustering scheme for cgMLST (HierCC) and the *Salmonella* database’s cgMLST scheme consisting of 3002 loci.^39^ Phylogenetic dendrogram of pLS20223695_NDM5, pLS20223694_NDM5 and publicly available similar plasmids was constructed by using RAxML software (http://sco.h-its.org/exelixis/web/software/raxml/) with the maximum likelihood method.

#### Conjugation experiments

*S.* Typhimurium LS20223695 and *E. coli* LS20223694 were used as the donor strains and sodium-azide-resistant *E. coli* J53 was used as the recipient strain to evaluate the transferability of the *bla*_NDM-5_ bearing plasmid. Briefly, overnight incubation of *S.* Typhimurium LS20223695, *E. coli* LS20223694 and J53 were blended with Luria-Bertani broth (LB) (Hangzhou Binhe Microorganism Reagent Co., Ltd) at 37°C for 4 h shaking until the logarithmic phase was reached. The donor and the recipient (LS20223695 and J53 or LS20223694 and J53) were mixed at a ratio of 1:1 in LB agar media. After incubation for 24 h at 37°C, the bacterial admixture was disseminated on tryptic soy agar (TSA) (Hangzhou Binhe Microorganism Reagent Co., Ltd) supplemented with sodium azide (200 μg/mL) and meropenem (0.5 μg /mL) for the selection of transconjugants. The transconjugants were then identified via antimicrobial susceptibility tests and WGS as previously described. The conjugation rates equaled to the number of transconjugants divided by the number of recipients.

#### S1-PFGE and southern hybridization

S1-Pulsed field gel electrophoresis (S1-PFGE) and Southern blotting were conducted to reveal the physical location of the *bla*_NDM-5_ determinant as described previously.^40^ Briefly, overnight cultures of *S.* Typhimurium LS20223695, *E. coli* LS20223694 and *E. coli* strains (LS2023660 and LS2023718) were imbedded in 1.0% agarose gel plugs, which was followed by the protease K treatment. S1-nuclease (Takara, Dalian, China) was used to treat agarose gel plugs, prior to the separation of DNA fragments by PFGE. Then, Southern hybridization was routinely conducted according to the manufacturer’s protocol, using the digoxigenin-labeled PCR products targeting *bla*_NDM-5_.

### Quantification and statistical analysis

There are no quantification or statistical analyses to include in this study.
