# Supplementary material for: IS15DIV-flanked composite transposon harboring blaNDM-5 in multidrug-resistant Salmonella Typhimurium
Source: iScience. 2024 Dec 31;28(2):111720. doi: 10.1016/j.isci.2024.111720 (PMC11772981; doi:10.1016/j.isci.2024.111720)
Supplement: Document S1. Figure S1 [file mmc1.pdf]

iScience, Volume *28*

Supplemental information

IS*15DIV*-flanked composite transposon harboring *bla*$_{NDM-5}$ in multidrug-resistant *Salmonella* Typhimurium

Kaiting Zhao, Jing Jin, Yuan Liao, Aixia Liu, Wugao Liu, and Weiping Wu

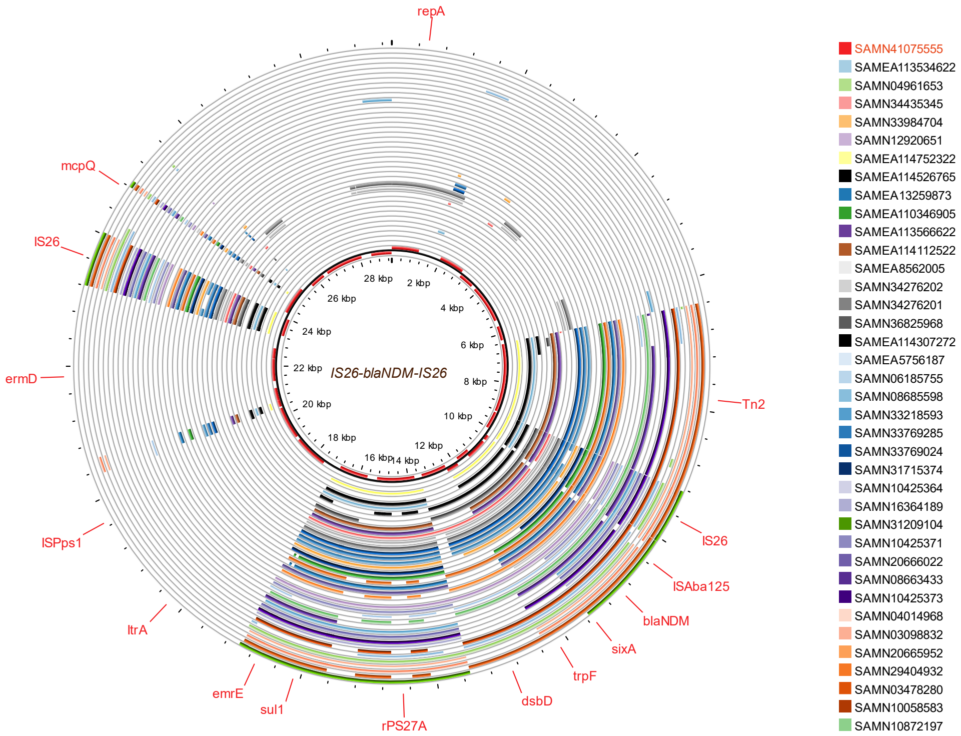

Figure S1. Circular alignment of IS*26*-*bla*$_{\mathrm{NDM}}$-IS*26* composite transposon between *S.* Typhimurium LS20223695 (BioSample ID: SAMN41075555) and 61 NDM-CRSE retrieved from GeneBank database, Related to Figure 1. SAMN41075555 recovered from this study are highlighted in red. **NDM-CRSE**: carbapenem-resistant *Salmonella enterica* harboring *bla*$_{\mathrm{NDM}}$. The repA, IS elements and resistance genes are annotated by red fonts.
